# Epstein–Barr Virus^+^ Smooth Muscle Tumors as Manifestation of Primary Immunodeficiency Disorders

**DOI:** 10.3389/fimmu.2018.00368

**Published:** 2018-02-27

**Authors:** Thomas Magg, Tilmann Schober, Christoph Walz, Julia Ley-Zaporozhan, Fabio Facchetti, Christoph Klein, Fabian Hauck

**Affiliations:** ^1^Department of Pediatrics, Dr. von Hauner Children’s Hospital, University Hospital, LMU Munich, Munich, Germany; ^2^Faculty of Medicine, Institute of Pathology, LMU Munich, Munich, Germany; ^3^Department of Radiology, University Hospital, LMU Munich, Munich, Germany; ^4^Department of Molecular and Translational Medicine, Pathology Unit, University of Brescia School of Medicine, Spedali Civili Brescia, Brescia, Italy

**Keywords:** Epstein–Barr virus, smooth muscle tumor, primary immunodeficiency disorder, secondary immuno-deficiency disorder, allogeneic hematopoietic stem cell transplantation, CARMIL2, GATA2

## Abstract

Epstein–Barr virus positive (EBV^+^) smooth muscle tumors (SMTs) constitute a very rare oncological entity. They usually develop in the context of secondary immunodeficiency caused by human immunodeficiency virus infection or immunosuppressive treatment after solid organ transplantation. However, in a small fraction of predominantly pediatric patients, EBV^+^ SMTs may occur in patients with primary immunodeficiency disorders (PIDs), such as GATA2 and CARMIL2 deficiency. In secondary immunodeficiencies and when the underlying condition can not be cured, the treatment of EBV^+^ SMTs is based on surgery in combination with antiretroviral and reduced or altered immunosuppressive pharmacotherapy, respectively. Importantly, without definitive reconstitution of cellular immunity, long-term survival is poor. This is particularly relevant for patients with EBV^+^ SMTs on the basis of PIDs. Recently, allogeneic hematopoietic stem cell transplantation resulted in cure of immunodeficiency and EBV^+^ SMTs in a GATA2-deficient patient. We propose that in the absence of secondary immunodeficiency disorders patients presenting with EBV^+^ SMTs should be thoroughly evaluated for PIDs. Allogeneic hematopoietic stem cell transplantation should be taken into consideration, ideally in the setting of a prospective clinical trial.

## Introduction

Epstein–Barr virus (EBV) is a gamma 1 herpes virus that preferentially infects human epithelial cells of the oropharynx and B cells of the adaptive immune system to establish lifelong latency (1). Rarely, EBV can cause ectopic infections and has been found in NK, T, gastric epithelial, and smooth muscle cells as well (1, 2). In the majority of cases, primary EBV infection is asymptomatic. However, EBV infection can cause lymphoproliferative phenotypes ranging from common infectious mononucleosis to rare hemophagocytic lymphohistiocytosis (3). Additionally, EBV infection presents as chronic active infection and is associated with autoimmune disorders, such as multiple sclerosis (4, 5).

Epstein–Barr virus has an inherent capacity of immortalization and malignant transformation especially of its B cell target (6). In the laboratory, this is used to generate lymphoblastoid cell lines. *In vivo*, this can lead to post transplant lymphoproliferative disorder (PTLD) and malignant lymphoma, such as Hodgkin’s lymphoma, Burkitt’s lymphoma, and diffuse large B cell lymphoma (7–9).

While the underlying conditions for the more frequent lymphoproliferative phenotypes seem to be heterogeneous and combinatorial, especially the rare and severe phenotypes are associated with secondary immunodeficiency disorders (SIDs) or predisposing genotypes, such as hemizygous *SH2D1A* mutations that cause X-linked lymphoproliferative syndrome (1, 3).

Smooth muscle tumors (SMTs) represent a heterogeneous group of disorders with a broad pathological spectrum ranging from very common and benign uterine leiomyoma to malignant leiomyosarcoma. The latter is characterized by hypercellularity, nuclear atypia, high mitotic rate, and tumor cell necrosis (10). EBV^+^ SMTs are a distinct subset of SMTs and have often been named leiomyoma or leiomyosarcoma because of their close histological appearance to common SMTs. EBV^+^ SMTs are very rare and can be encountered at any age in the context of SIDs and in a small fraction of predominantly pediatric patients with primary immunodeficiency disorders (PIDs) (2, 11).

Here, we review the current knowledge on EBV^+^ SMTs in general and present it as an emerging manifestation of PIDs that might be targeted by allogeneic hematopoietic stem cell transplantation (alloHSCT).

## Presentation, Pathogenesis, and Treatment of EBV^+^ SMTs

**Figure 1:**
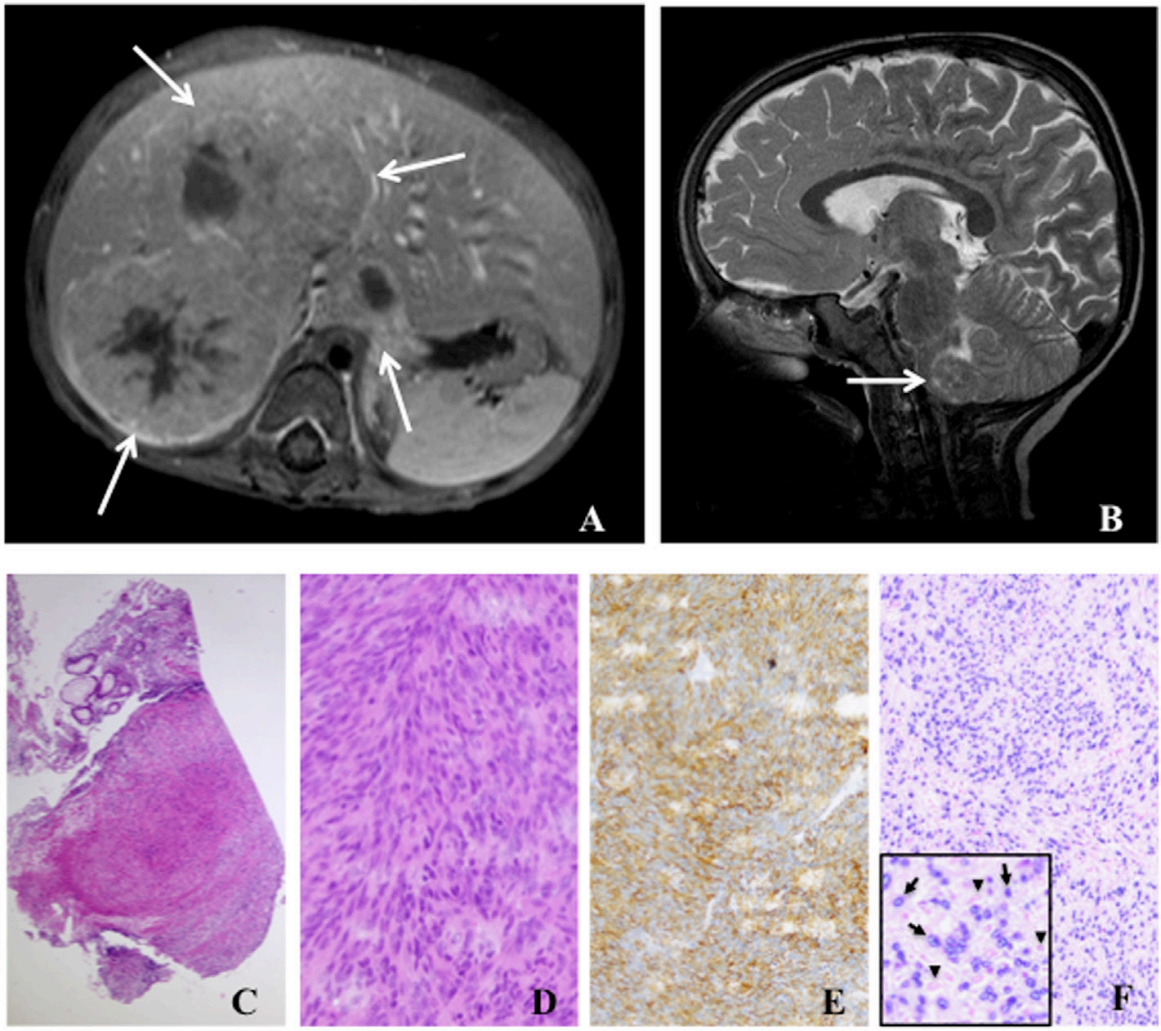
Radiology and histology of Epstein–Barr virus positive (EBV^+^) smooth muscle tumors. **(A)** Abdominal magnetic resonance image (T1 fat-sat post contrast medium) shows solid liver tumors involving segments I and V–VIII (arrows). **(B)** Cranial magnetic resonance image (T2 sagittal) displays a tumor in the medulla oblongata (arrow). **(C)** Low-power (50×) examination of a colon biopsy shows a prominent nodular cellular proliferation in the mucosa and submucosa **(D)** High-power (400×) magnification displays fascicles of fusiform spindle cells with abundant eosinophilic cytoplasm and elongated or ovoid nuclei without significant atypia or mitoses suggesting a mesenchymal neoplasia of smooth muscle origin **(E)**. Immunohistochemistry for smooth muscle actin (200×) confirms the smooth muscle nature of the tumor **(F)**. EBV association is demonstrated by *in situ* hybridization for EBV-encoded RNA (EBER) in the same lesion (200×). Inlet displays positive cells with EBER in darkly stained basophilic nuclei (arrows) and negative cells with faint eosinophilic nuclei (arrowheads).

Most EBV^+^ SMTs develop at any age in patients with SIDs due to uncontrolled human immunodeficiency virus infection (HIV EBV^+^ SMTs) and organ transplantation-associated immunosuppressive treatment (PT EBV^+^ SMTs) (2, 12). Additionally, they rarely present in pediatric patients with proven or suspected PID (PID EBV^+^ SMTs) (11). Overall the prevalence of EBV^+^ SMTs is estimated to be <1–5% for each patient group. Particularly, in the PT EBV^+^ SMTs group they present as late complications (median 48 months, range 5–348 months) (12, 13). The clinical manifestation of EBV^+^ SMTs is unspecific and mainly depends on the tumor localization, the tumor size, and the particular organ displacement and/or disruption (12, 13). The majority of EBV^+^ SMTs is located in the liver, but virtually every other organ can be affected and frequently the lungs, the gastrointestinal tract, the central nervous system, and the adrenal glands are involved (Figures 1A,B) (12, 13). Importantly, radiological imaging can not establish the diagnosis of EBV^+^ SMTs as there are no pathognomonic findings. EBV^+^ SMTs can occur at single or multiple sites synchronously or metachronously, grow *per continuitatem* and do not metastasize (12, 13). They are believed to originate from myogenous venous wall cells and can be of recipient or donor origin in the setting of solid organ transplantation (12, 14, 15). Molecular genetic studies have shown that multiple EBV^+^ SMTs frequently constitute independent clones rather than metastases of a single tumor (16).

EBV^+^ SMTs have a spindle-shaped cell morphology with eosinophilic cytoplasm and elongated nuclei, but frequently lack histological atypia, elevated mitotic activity, and tumor necrosis (Figures 1C,D). Especially in association with HIV they can present with sarcoma-like histological features and frequently infiltrating lymphocytes and histiocytes can be detected. Expression of smooth muscle differentiation markers, such as smooth muscle actin, caldesmon, vimentin, and desmin, and transcriptional activity of EBV are demonstrated by immunohistochemistry and EBV-encoded small RNA (EBER) *in situ* hybridization (ISH), respectively (Figures 1E,F). Immunohistochemistry alone can lead to false negative results (17, 18). Importantly, histopathology findings do neither correlate with tumorbiology nor disease activity, but are mandatory to establish the diagnosis of EBV^+^ SMTs (11, 12).

In general in EBV^+^ SMTs, EBV achieves a latency type III-like pattern, i.e., cells are positive for EBV nuclear antigen 2 (EBNA2), EBNA3, and late membrane protein 1 (19–22). The majority of HIV EBV^+^ SMTs are positive for complement receptor 2 (CR2 or CD21) that is bound by EBV during B cell infection, while a substantial number of PT EBV^+^ SMTs and all analyzed cases of PID EBV^+^ SMTs are CD21 negative (21, 23, 24). Thus, the precise EBV entry mechanism into the tumor progenitor cells is unknown. It is possible that several entry routes exist. Also the molecular pathophysiology of tumorigenesis remains poorly understood. The activated mTOR/AKT-pathway seems to be involved and increased v-myc expression has been found (12, 25, 26).

The diagnosis of EBV^+^ SMTs is suspected in the context of SID and PID and, because there is no pathognomonic radiological morphology, biopsy based histopathology, immunohisto-chemistry, and EBER ISH are mandatory to formally establish the diagnosis (13).

The treatment of EBV^+^ SMTs is based on the principle of re-establishing efficient T cell immunity. In patients with HIV infection, appropriate antiretroviral treatment should be given. Patients with iatrogenic immunosuppression following organ transplantation may benefit from reduction of immunosuppressive treatment. It remains a matter of debate whether switching immunosuppression toward a mTOR inhibitor, such as sirolimus might lead to a more favorable outcome (27). Surgery should be performed whenever tumor masses compromise organ functions. Chemotherapy and radiotherapy can be applied but in general neither of these approaches is markedly improving the disease course (13). Prognostic data are derived from retrospective analyses of case records and show a five-year overall survival (OS) of approximately 50% for HIV EBV^+^ SMTs and PT EBV^+^ SMTs, while OS of PID EBV^+^ SMTs tends to be 0% (13). Especially, multiorgan involvement (*n* = 33/68, OS = 48.5%) and intracranial manifestations (*n* = 7/68, OS = 10%) are contributing to the dismal prognosis (13).

## PIDs Underlying EBV^+^ SMTs

The first description of PT EBV^+^ SMTs dates back to 1970, but it was not until 1995 that the first systematic studies on HIV EBV^+^ SMTs and PID EBV^+^ SMTs were published (2, 28, 29). Up to date only very few cases of PID EBV^+^ SMTs have been reported and a substantial proportion of these patients lack a precise molecular PID diagnosis (Table 1) (19–21, 30–37).

**Table 1:** Epstein–Barr virus positive (EBV^+^) smooth muscle tumors (SMTs) in patients with primary immunodeficiency disorders (PIDs).

Publication	No. patient	Reported histology^a^	Reported type of PID (*Gene*)	Preceeding stem cell transplantation	SMT location	EBV viremia	PTLD	Treatment	Outcome
Mierau et al. (31)	1	Leiomyosarcoma	Common variable immunodeficiency	No	Brain	n.a.	No	Surgery	Tumor-free for 18 months

Tulbah et al. (36)	1	Leiomyosarcoma	Congenital T cell immunodeficiency	No	Thyroid, liver and lung	n.a.	No	Unclear	Lost to follow up

Reyes et al. (20)	1	Leiomyosarcoma	Ataxia telangiectasia (*ATM*)	No	Larynx, small bowel	n.a.	No	Surgery	Not reported

Monforte-Muñoz et al. (32)	1	Leiomyomatosis	Severe combined immunodeficiency (SCID) (*ADA*)	Yes	Gall bladder, liver, spleen, pancreas, intestinal tract and lung	n.a.	Yes	Unclear	Unclear

Hatano et al. (19)	1	Leiomyoma	Cellular and complement immunodeficiency	No	Lung	n.a.	No	Surgery	Tumor-free for >2 years

Atluri et al. (30)	1	Leiomyomatosis	SCID (*IL2RG*)	Yes	Lung, bilateral renal	Negative	Yes	Donor lymphocyte infusion	Tumor stable for >2 years

Vinh et al (37)	1	Leiomyosarcoma	GATA2 haploinsufficiency (*GATA2*)	No	Orbit, liver, colon, and uterus	n.a.	No	Surgery and stem cell transplantation	Died of post transplant viral infections

Shaw et al. (35)	1	Smooth muscle tumor	NK cell deficiency	No	Bilateral adrenal	n.a.	No	Surgery	Tumor-free for 26 months

Petrilli et al. (34)	1	Smooth muscle tumor	SCID (*ZAP70*)	No	Bilateral adrenal	n.a.	Yes	Surgery and stem cell transplantation	Died of EBV^+^ multifocal diffuse large B cell lymphoma five years after unsuccessful allogeneic hematopoietic stem cell transplantation (alloHSCT)

Parta et al. (33)	1	Smooth muscle tumor	GATA2 haploinsufficiency (*GATA2*)	No	Liver, vertebral	Positive	No	Stem cell transplantation	Cured with a three year follow up after alloHSCT

Schober et al. (21)	4	Smooth muscle tumor	CID (*CARMIL2*)	No	Gut, liver, lung, spleen, kidney, brain	Positive in 1/4	No	Surgery and chemotherapy	Died of EBV^+^ SMT-induced multi-organ failure

*^a^EBV^+^ SMTs were originally divided into EBV-associated leiomyomas and leiomyosarcomas, but current classification holds them all collectively as EBV^+^ SMTs*.

The first report by Mierau et al. dates to 1997 and describes a 14-year-old female with primary leiomyosarcoma of the brain in the context of common variable immunodeficiency (CVID) (31). The authors emphasize the need for proper histopathological work-up of unusual tumor entities in immunocompromised patients. In view of a positive family history, the authors conclude that EBV^+^ SMTs are caused by an inherited rather than acquired disorder (31).

In 1999, Tulbah and colleagues published another case of a child with a genetically undefined congenital immunodeficiency presenting with multifocal EBV^+^ SMTs located to the thyroid gland, liver, and lung, and stated that they are unaware of comparable cases (36).

The first report of a genetically proven PID, namely ataxia telangiectasia (AT), associated with EBV^+^ laryngeal leiomyosarcoma and jejunal cellular leiomyoma is published by Reyes et al. in 2002 (20). The authors conclude that EBV^+^ SMTs are related to the immunosuppressive consequences of AT and that searching for infectious causes is important as SMTs have been reported in AT without subsequent evaluation of underlying EBV infection (20).

In 2003, Monforte-Muñoz et al. published the case of an 8-year-old female with severe combined immunodeficiency (SCID) caused by adenosine deaminase (ADA) deficiency. The patient develops EBV^+^ SMTs in the gallbladder, spleen, pancreas, intestinal tract, and lung after alloHSCT. Additionally, the patient presents with EBV^+^ PTLD, pulmonary and gastric adenovirus, and large intestine cryptosporidum infections all of which are indicative of poor immune reconstitution and/or recurrences of the ADA-SCID (32). The authors state that the occurrence of EBV^+^ SMTs and EBV^+^ PTLD suggests a common pathogenesis that may have therapeutic and prognostic implications (32).

In 2006, Hatano et al. reported a 6-year-old male with an EBV^+^ SMT in the right bronchus that leads to atelectasis and abscess in the right upper and middle lobe (19). They find reduced numbers of T cells and impaired T cell proliferation after stimulation with phytohemagglutinin. As the patient has additional recurrent infections, they conclude that he has an undefined cellular immunodeficiency (19). We are currently investigating the precise molecular cause of the suspected PID.

In 2007, Atluri et al. published an *IL2RG* SCID patient who is treated with haploidentical alloHSCT and 8 years thereafter presents with renal and pulmonary EBV^+^ SMTs in the context of mixed donor T cell chimerism (30). Importantly, after donor lymphocyte infusion the EBV^+^ SMTs rest stable during a 2-year follow up and the authors conclude that EBV^+^ SMTs after partial immunoreconstitution may not require surgery or chemotherapy (30).

In 2012, Shaw et al. reported a 12-year-old female with quantitative classic NK cell deficiency presenting with bilateral adrenal EBV^+^ SMTs that are treated by successful surgery with an event free follow up of 26 months (35). They perform intensive immunological analysis and document a marked deficiency of absolute numbers and cytotoxicity of CD3^−^CD16^+^CD56^+^ NK cells at four separate timepoints over 18 months. The authors do not report an underlying genetic condition, but as NK cells are known to participate in protective EBV immunity, they speculate that the severe NK cell deficiency contributes to the development of EBV^+^ SMTs (35).

In 2014, Petrilli et al. reported a 7-year-old female with bilateral adrenal EBV^+^ SMTs and as the patient has recurrent respiratory tract infections, including tuberculosis, reduced immunoglobulins, and impaired T cell proliferation after mitogenic stimulation, they perform alloHSCT. Five years after unsuccessful alloHSCT, the patient develops lethal EBV^+^ multifocal diffuse large B cell lymphoma (34). The two different tumor entities are caused by independent EBV transformations and the EBV^+^ SMTs infiltrating lymphocytes are predominantly CD3^+^CD5^+^CD8^+^ T cells (34). In 2016 and in collaboration with Petrilli and coworkers we find a homozygous autosomal recessive mutation in *ZAP70* (c.1765G > A, p.Val589Met) that could explain the patient’s PID phenotype and would modify the diagnosis into (CID, Schober et al., unpublished data).

GATA2 haploinsufficiency is a recently identified polymorphic PID that manifests with a variety of infectious complications especially caused by mycobacteria, but as well by viral, bacterial, and fungal pathogens (38). In 2010, Vinh et al reported a 41-year-old female with multiple infections in the context of GATA2 haploinsufficiency and EBV^+^ SMTs located to the orbit, liver, colon, and uterus. The patient is treated with alloHSCT, but succumbs to post transplant viral infections (37).

In 2016, Parta et al. reported a 24-year-old male with GATA2 haploinsufficiency causing a polymorphic PID including EBV^+^ SMTs of the liver and possibly the spleen and the bones (33). They perform alloHSCT with a myeloablative conditioning regimen and peripheral blood hematopoietic stem cells from a matched sibling donor. After a 3-year follow up GATA2 haploinsufficiency and EBV^+^ SMTs are resolved and the authors conclude that at least in the context of GATA2 haploinsufficiency alloHSCT can lead to reconstitution of immunologic function and thereby cure of EBV-associated malignancy (33).

In 2017, our group reports four patients with EBV^+^ SMTs on the background of a novel CID caused by homozygous autosomal recessive *CARMIL2* mutations (21). Two of the patients initially are reported as cases of infantile myofibromatosis, but extensive immunobiological analyses reveal a profound regulatory T cell deficiency, defective CD28 co-signaling associated with impaired T cell activation, differentiation and function, as well as perturbed cytoskeletal organization associated with T cell polarity and migration disorders (21, 39, 40). Two patients decease before the PID diagnosis is established and the other two patients succumb to disease complications while being prepared for alloHSCT (21).

In summary, we are aware of 14 PID cases with EBV^+^ SMTs (Table 1). Twelve of them develop EBV^+^ SMTs as a primary PID manifestation and two of them develop the tumors after alloHSCT. Four of the cases are published without and ten with a genetic diagnosis confined to the *ATM, ADA, IL2RG, GATA2*, and *CARMIL2* genes. In one of the genetically undefined cases, we are able to retrospectively identify a mutation in the *ZAP70* gene. All of the genetically and immunologically defined PIDs impair T and/or NK cell immunity. Four of the reported cases are treated with alloHSCT in the presence of EBV^+^ SMTs and two of them decease because of viral infections or B cell lymphoma, while one develops stable disease after donor lymphocyte infusion and one is cured from PID and EBV^+^ SMTs.

## Conclusion

EBV^+^ SMTs constitute very rare tumors seen in the context of SIDs caused by human immunodeficiency virus infection or immunosuppressive treatment after solid organ transplantation (11). The pathogenesis of EBV^+^ SMTs remains largely unknown, but it is evident that an immunocompromised host is a *conditio sine qua non* and that especially T and NK cell immunity is important to prevent the disease (11, 21, 35). Later, EBV^+^ SMTs emerge as possible manifestations of PIDs and up to now have been linked to mutations in *ATM, ADA, IL2RG, ZAP70, GATA2*, and *CARMIL2* (19–21, 30, 32–34, 37). Additionally, they have been found in genetically undefined PIDs and based on the clinical and immunological findings and our growing understanding of their pathogenesis these PIDs should at best be named CID and classic NK cell deficiency (31, 35, 36). At present, a particular molecular signaling or effector pathway has not been identified as a prerequisite to develop EBV^+^ SMTs. Given the multitude of CID causing gene defects, we thus propose an unbiased genetic work-up, such as whole exome sequencing to search for molecular PID causes in patients with EBV^+^ SMTs of unknown origin (41). In order to treat EBV^+^ SMTs, whenever possible, HIV infection should be addressed and post transplant immunosuppressive treatment should be reduced. AlloHSCT is a well-established curative treatment for CID and other PID and, therefore, seems a promising therapeutic approach for PID that is present with EBV^+^ SMTs (13, 33, 42).

Four major conclusions can be drawn at present. First, unusual SMTs should be screened for the presence of EBV preferentially by using EBER ISH. Second, in patients presenting with EBV^+^ SMTs without obvious SIDs, PIDs have to be considered strongly, necessitating appropriate investigation. Third, in PID patients presenting with solid tumors, EBV^+^ SMTs are a differential diagnosis. Fourth, PID patients manifesting with EBV^+^ SMTs might be treated with up front alloHSCT ideally in the setting of a prospective clinical trial yet to be initiated.

We envision that with increased awareness toward EBV^+^ SMTs as a manifestation of PIDs the rate of proper diagnosis of this association will increase and the outcome of curative alloHSCT will improve.

## Author Contributions

TM and FH wrote the article. TS, FF, and CK revised the article. CW provided histopathological images. JL-Z provided radiological images.

## Conflict of Interest Statement

The authors declare that the research was conducted in the absence of any commercial or financial relationships that could be construed as a potential conflict of interest.
